# Na_V_1.8 as Proarrhythmic Target in a Ventricular Cardiac Stem Cell Model

**DOI:** 10.3390/ijms25116144

**Published:** 2024-06-02

**Authors:** Nico Hartmann, Maria Knierim, Wiebke Maurer, Nataliya Dybkova, Florian Zeman, Gerd Hasenfuß, Samuel Sossalla, Katrin Streckfuss-Bömeke

**Affiliations:** 1 Clinic for Cardiology and Pneumology, University Medical Center, 37075 Göttingen, Germany; 2 DZHK (German Center for Cardiovascular Research), Partner Site Göttingen and Rhein Main, 61231 Bad Nauheim, Germany; 3 Clinic for Cardio-Thoracic and Vascular Surgery, University Medical Center, 37075 Göttingen, Germany; 4 Center for Clinicial Trials, University of Regensburg, 93042 Regensburg, Germany; 5 Medical Clinic I, Cardiology and Angiology, Giessen and Department of Cardiology at Kerckhoff Heart and Lung Center, Justus-Liebig-University, 61231 Bad Nauheim, Germany; 6 Institute of Pharmacology and Toxicology, University of Würzburg, 97078 Würzburg, Germany

**Keywords:** Na_V_1.8, *SCN10A*, ventricular arrhythmias, induced pluripotent stem cells, CRISPR Cas9, ventricular cells

## Abstract

The sodium channel Na_V_1.8, encoded by the *SCN10A* gene, has recently emerged as a potential regulator of cardiac electrophysiology. We have previously shown that Na_V_1.8 contributes to arrhythmogenesis by inducing a persistent Na^+^ current (late Na^+^ current, I_NaL_) in human atrial and ventricular cardiomyocytes (CM). We now aim to further investigate the contribution of Na_V_1.8 to human ventricular arrhythmogenesis at the CM-specific level using pharmacological inhibition as well as a genetic knockout (KO) of *SCN10A* in induced pluripotent stem cell CM (iPSC-CM). In functional voltage-clamp experiments, we demonstrate that I_NaL_ was significantly reduced in ventricular *SCN10A*-KO iPSC-CM and in control CM after a specific pharmacological inhibition of Na_V_1.8. In contrast, we did not find any effects on ventricular APD_90_. The frequency of spontaneous sarcoplasmic reticulum Ca^2+^ sparks and waves were reduced in *SCN10A-*KO iPSC-CM and control cells following the pharmacological inhibition of Na_V_1.8. We further analyzed potential triggers of arrhythmias and found reduced delayed afterdepolarizations (DAD) in *SCN10A-*KO iPSC-CM and after the specific inhibition of Na_V_1.8 in control cells. In conclusion, we show that Na_V_1.8-induced I_NaL_ primarily impacts arrhythmogenesis at a subcellular level, with minimal effects on systolic cellular Ca^2+^ release. The inhibition or knockout of Na_V_1.8 diminishes proarrhythmic triggers in ventricular CM. In conjunction with our previously published results, this work confirms Na_V_1.8 as a proarrhythmic target that may be useful in an anti-arrhythmic therapeutic strategy.

## 1. Introduction

Cardiac electrophysiology is a complex and precisely regulated process crucial for the normal functioning of the heart. Sodium channels are essential for the rapid depolarization phase of the cardiac action potential (AP) and contribute to initiating and propagating electrical signals [1,2]. However, under certain pathological conditions, such as heart failure (HF), ischemia, and atrial fibrillation (AF), voltage-gated (Na_V_) sodium channels exhibit increased reactivation or sustained opening, resulting in the relevant augmentation of a persistent inward current known as the late sodium current (I_NaL_) [3,4,5,6,7,8,9,10,11]. One particular sodium channel isoform, Na_V_1.8, encoded by the *SCN10A* gene, has gained attention due to its potential involvement in cardiac electrophysiology. Extensive research has shown that Na_V_1.8 contributes to nociceptive signaling and pain perception in peripheral sensory neurons [12,13,14,15]. However, genome-wide association studies reported that the *SCN10A* gene is associated with cardiac arrhythmias [16,17,18]. Although several studies have investigated the expression of *SCN10A* in ventricular myocardium [19,20,21,22,23,24], there is an ongoing debate regarding the mechanism responsible for the effects associated with Na_V_1.8 and the specific cellular localization of these effects, whether it is in cardiac ganglia or cardiomyocytes (CM) [20,23]. The present study addresses this knowledge gap using genetic ablation or knockout (KO) of *SCN10A* in human induced pluripotent stem cell-CM (iPSC-CM) as a tool to supplement the use of pharmacological agents that inhibit the channel. This approach can reveal the inherent impact of Na_V_1.8 on I_NaL_ in human CM without any confounding or unspecific effects of pharmacological inhibitors. This *SCN10A* iPSC-CM KO model was used in our previous study and demonstrated that Na_V_1.8 is expressed in HF CM and contributes to I_NaL_ formation in ventricular iPSC-CM [22]. Since it is still unclear how Na_V_1.8-dependent I_NaL_ contributes to cellular arrhythmias, the objective of this study was to investigate potential triggers of arrhythmias in human ventricular CM. Therefore, we conducted additional cellular electrophysiological measurements, including the spontaneous release of Ca^2+^ from the sarcoplasmic reticulum (SR), action potential duration (APD), delayed afterdepolarizations (DADs), and Ca^2+^ transients to further characterize the arrhythmogenic potential of Na_V_1.8 in the heart. By utilizing CRISPR/Cas9-based KO technology, we provide compelling evidence for the existence of a Na_V_1.8-mediated I_NaL_ in human CM, contributing valuable information to the existing body of knowledge [22].

## 2. Results

### 2.1. Effects of Na_V_1.8 on I_NaL_ in Human Ventricular iPSC-Cardiomyocytes

**Figure 1:**
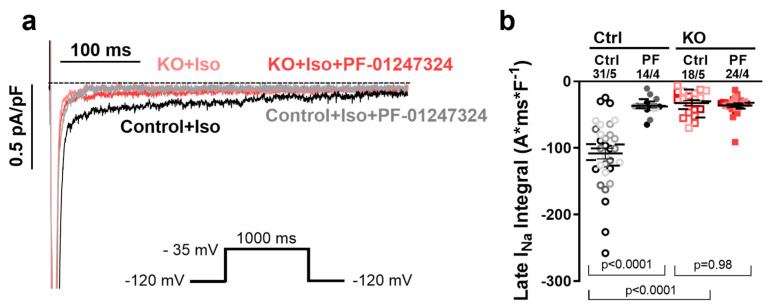
**Na_V_1.8 contributes to I_NaL_ formation in ventricular iPSC-CM.** (**a**) Original traces of I_NaL_ in control iPSC-CM vs. SCN10A KO-iPSC-CM cells according to the inserted protocol. (**b**) Mean values per differentiation ± SEM of I_NaL_ (control n = 31 cells/5 differentiations; control + PF n = 14 cells/4 differentiations; SCN10A KO iPSC-CM control n = 18 cells/5 differentiations, SCN10A KO-iPSC-CM + PF n = 24 cells/4 differentiations). Single cells are presented as individual symbols and different iPSC-CM differentiations are color-coded. The small horizontal lines indicate the mean values per differentiation. Data were compared using nested one-way ANOVA with Sidak’s test for multiple comparisons to calculate *p* values.

Homozygous *SCN10A*/Na_V_1.8 KO iPSC were differentiated into 2-month-old ventricular CM as described elsewhere [25]. In our previous study, we demonstrated that the knockout of Na_V_1.8 in ventricular iPSC-CM after mild isoproterenol stimulation (Iso, 50 nmol/L) resulted in a substantial reduction (~70%) in the amplitude of I_NaL_ compared to control iPSC-CM [22]. The application of a Na_V_1.8-specific blocker (PF-01247324) did not produce any additional effect on I_NaL_ in Na_V_1.8 KO iPSC-CM compared to untreated Na_V_1.8 knockout cells [22]. In the present study, we aimed to validate and expand upon these initial findings by conducting a larger-scale investigation involving a higher number of single ventricular iPSC-CM generated from a higher number of cardiac differentiations. To increase the relatively small amplitude of I_NaL_ in healthy human iPSC-CM, we used 50 nmol/L Iso, which is consistent with our previous experiments. To assess potential non-specific effects associated with either genetic KO or pharmacological intervention, we utilized the specific Na_V_1.8 blocker PF-01247324 at a concentration of 1 µmol/L [22]. In functional voltage-clamp experiments, we showed that I_NaL_ was significantly reduced by pharmacological inhibition and the genetic KO of Na_V_1.8, validating the impact of both approaches on I_NaL_ (Figure 1). The increased I_NaL_ induced by Iso in control ventricular iPSC-CM (−108.4 ± 9.5 A*ms*F^−1^) was significantly reduced in KO iPSC-CM by 70.8% (−32.5 ± 4.4 A*ms*F^−1^, *p* < 0.0001, Figure 1b) and by the specific Na_V_1.8 inhibitor, PF-01247324 (1 µmol/L), by 70.0% (−31.6 ± 6.7 A*ms*F^−1^; *p* < 0.0001; Figure 1b). No further effects on I_NaL_ were observed in KO iPSC-CM after the application of PF-01247324 (−36.3 ± 3.0 A*ms*F^−1^; *p* < 0.981; Figure 1b). These results demonstrated in a broader set of experiments based on statistical analyses of multiple cardiac differentiations and not on single iPSC-CM, confirm our previous results that Na_V_1.8 contributes to I_NaL_ formation in ventricular iPSC-CM, demonstrated in a broader set of experiments based on statistical analyses of multiple cardiac differentiations and not single iPSC-CM.

### 2.2. Effects of Na_V_1.8 on APD and Arrhythmic Triggers in Ventricular Cells

**Figure 2:**
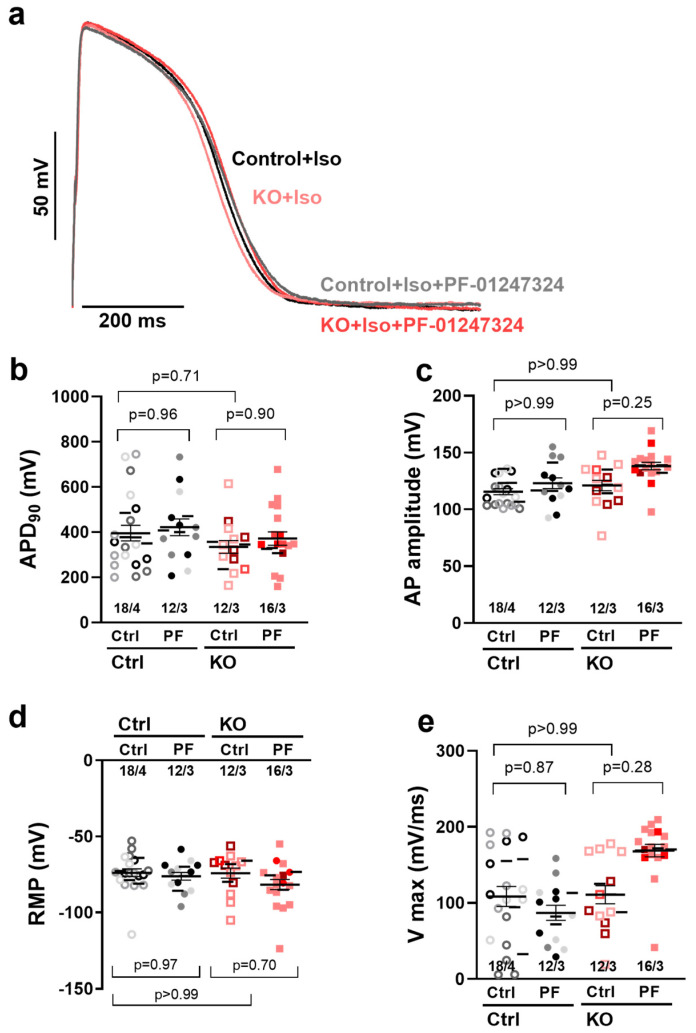
**Na_V_1.8 inhibition or KO has no significant impact on action potential parameters of iPSC-CM.** (**a**) Original traces of APD_90_ in ventricular control iPSC-CM vs. *SCN10A*-KO iPSC-CM at 0.5 Hz. Mean data ± SEM of (**b**) APD_90_, (**c**) AP amplitude, (**d**) RMP and (**e**) upstroke velocity (Vmax) of control and *SCN10A*-KO iPSCM (control n = 18 cells/4 differentiations; control + PF-01247324 [PF] n = 12 cells/3 differentiations; *SCN10A*-KO control n = 12 cells/3 differentiations, *SCN10A*-KO + PF n = 16 cells/3 differentiations). Single cells are presented as individual symbols and different iPSC-CM differentiations are color-coded. The small horizontal lines indicate the mean values per differentiation. Data were compared using nested one-way ANOVA with Sidak’s test for multiple comparisons to calculate *p* values.

To explore the potential effects of Na_V_1.8 on the action potential in human ventricular iPSC-CM, we conducted current-clamp experiments on whole cells. The APD of KO iPSC-CM was not significantly different from that of control cells, both with and without additional application of the specific Na_V_1.8 blocker PF-01247324 (Figure 2a,b, control at 0.5 Hz, APD_90_, 395.4 ± 42.0 ms vs. control + PF-01247324 420.9 ± 46.5 ms, an increase of 6%; KO control 340.4 ± 34.6 ms, a decrease of 13% vs. KO + PF-01247324 376.4 ± 34.8 ms, a reduction of 4.8%). Furthermore, the APD in KO cells at 20% repolarization (APD_20_), at 50% repolarization (APD_50_), and at 70% repolarization (APD_70_) were also similar to that of control cells (Supplemental Figure S1, Supplemental Table S1). Potential non-specific effects associated with KO or pharmacological inhibition of Na_V_1.8 were analyzed by comparing resting membrane potential (RMP), action potential amplitude (APA), and upstroke velocity (Vmax) between all groups. No significant effects of *SCN10A*-KO or pharmacological inhibition of Na_V_1.8 were observed on these parameters. 

### 2.3. Role of Na_V_1.8 in the Generation of SR Ca^2+^ Leak in the Ventricle

It is known that I_NaL_ can induce arrhythmogenic diastolic SR Ca^2+^ release [26]. Furthermore, we demonstrated in our previous studies that the arrhythmogenic potential of Na_V_1.8 in the ventricle is exerted through the enhancement of I_NaL_ [22,23] and, consequently, the induction of spontaneous SR Ca^2+^ release on a subcellular level. To further investigate these effects on a standardized larger scale at the CM-specific level and ultimately by KO of Na_V_1.8 in human CM, we conducted confocal microscopy experiments in ventricular human control and Na_V_1.8 KO iPSC-CM. 

**Figure 3:**
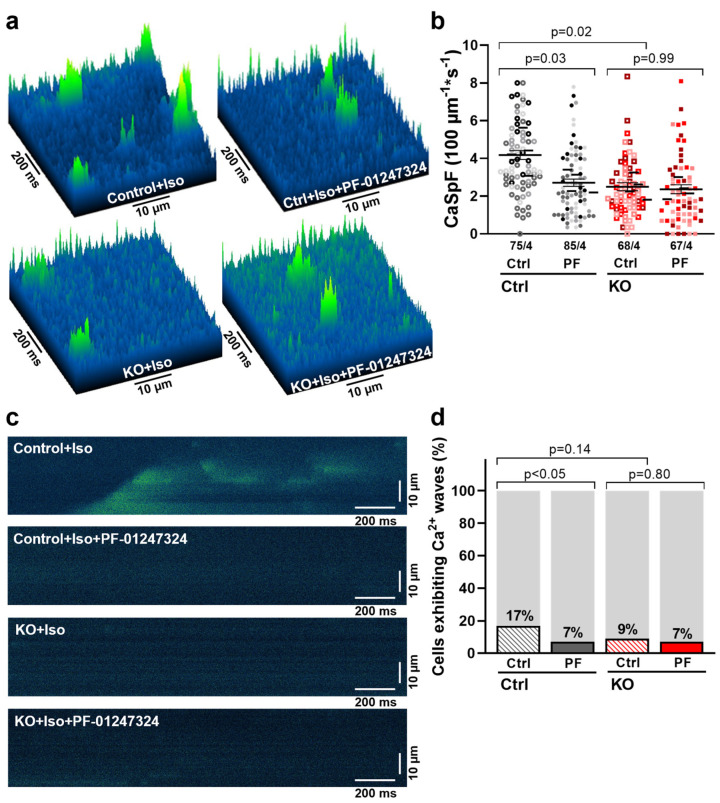
**Contribution of Na_V_1.8 to spontaneous diastolic SR Ca^2+^ release in ventricular iPSC-CM.** (**a**) Representative surface plots showing spontaneous diastolic Ca^2+^ sparks (green) in ventricular iPSC-CM. (**b**) Mean values of Ca^2+^ spark frequency (CaSpF) normalized to scan width and duration. Numbers indicate the total cell count of control CM (control, n = 75 cells/4 differentiations), control cells treated with Na_V_1.8 inhibitor PF-01247324 (control + PF-01247324 [PF], n = 85/4), and KO of Na_V_1.8 after control treatment and treatment with PF-01247324 (n = 68/4 vs. 67/4). (**c**) Original representative line-scans of ventricular iPSC-CM illustrating a spontaneous proarrhythmogenic diastolic Ca^2+^ wave (green). (**d**) Percentage of cells exhibiting diastolic Ca^2+^ waves (control: 17% vs. control + PF-01247324: 7% of cells exhibiting Ca^2+^ waves, *p* < 0.05; KO: 9% vs. KO + PF-01247324: 7%). Values are presented as mean ± SEM or absolute numbers. Single cells are presented as individual symbols and different iPSC-CM differentiations are color-coded. The small horizontal lines indicate the mean values per differentiation. Data were compared using nested one-way ANOVA with Sidak’s test for multiple comparisons to calculate *p* values. Proportions were compared using Fisher’s exact test.

Diastolic confocal line scans using Fluo 4-AM revealed that KO of *SCN10A* in ventricular human iPSC-CM resulted in a decrease in the frequency of spontaneous arrhythmogenic Ca^2+^ sparks compared to the corresponding control cells (control: 4.18 ± 0.25 vs. KO: 2.72 ± 0.19 sparks/100 µm/s, *p* = 0.02). When Na_V_1.8 was pharmacologically inhibited using PF-01247324, a significant reduction in diastolic Ca^2+^ sparks was observed in control cells (control + PF-01247324: 2.50 ± 0.22, *p* = 0.03) but not in KO cells (KO + PF-01247324: 2.36 ± 0.25, *p* = 0.99) (Figure 3a,b). Thus, the reduction in both I_NaL_ and diastolic SR Ca^2+^ release in Na_V_1.8 KO or after Na_V_1.8 inhibition confirms a significant role of this sodium channel in cellular arrhythmogenesis in the human ventricle.

However, the amount of systolic Ca^2+^ release and the dynamics of Ca^2+^ sequestration, as investigated in epifluorescence experiments using Fura 2-AM, were left unaffected by KO or pharmacological inhibition of Na_V_1.8 (Supplemental Figure S2). 

We further investigated the effects of pharmacological inhibition and genetic ablation of Na_V_1.8 on the incidence of diastolic Ca^2+^ waves, which constitute primary proarrhythmic triggers. The percentage of cells exhibiting diastolic Ca^2+^ waves was significantly reduced in control ventricular iPSC-CM by inhibition of Na_V_1.8 with PF-01247324 (percent of all cells exhibiting Ca^2+^ waves: control 17% vs. control + PF-01247324 7% of all cells exhibiting Ca^2+^ waves, *p* < 0.05). Genetic ablation of Na_V_1.8 showed a slight tendency to reduce Ca^2+^ wave frequency, but the effect was not statistically significant (control: 17% vs. KO: 9%; *p* = 0.14; Figure 3c,d).

### 2.4. Na_V_1.8 Inhibition or KO Reduces Proarrhythmic Triggers in Ventricular Cardiomyocytes

**Figure 4:**
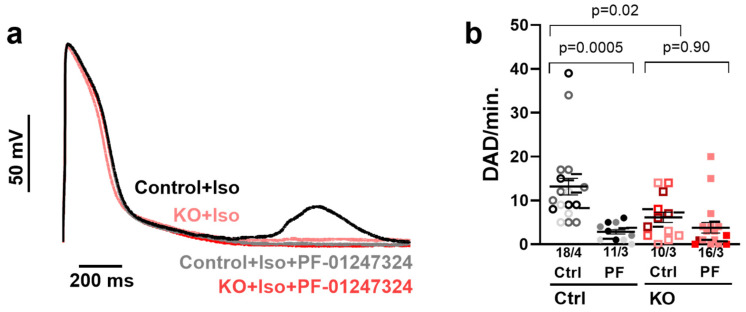
**Na_V_1.8 KO and inhibition reduce cellular arrhythmogenic triggers** (**a**) Original traces of DADs and triggered activity in control iPSC-CM vs. *SCN10A*-KO iPSC-CM at 0.5 Hz. (**b**) Mean values ± SEM of DAD/min. (control n = 18 cells/4 differentiations; ventricular control + PF n = 11 cells/3 differentiations; *SCN10A*-KO control n = 10 cells/3 differentiations, *SCN10A*-KO + PF n = 16 cells/3 differentiations). Single cells are presented as individual symbols and different iPSC-CM differentiations are color-coded. The small horizontal lines indicate the mean values per differentiation. Data were compared using nested one-way ANOVA with Sidak’s test for multiple comparisons to calculate *p* values.

To gain further insights into the contribution of I_NaL_ to cellular arrhythmias, we investigated potential triggers of arrhythmias. Because afterdepolarizations occur infrequently under basal conditions, we augmented cellular SR Ca^2+^ stores with isoproterenol (Iso) to increase cellular arrhythmias [27]. Under these conditions, we observed a pronounced number of DADs in control iPSC-CM. This effect was significantly reduced by inhibiting Na_V_1.8 with PF-01247324 and by genetically depleting Na_V_1.8 (DADs/min., Figure 4a,b, at 0.5 Hz control iPSC-CM 12.9 ± 1.4 DADs/min.; control iPSC-CM + PF-01247324: 2.8 ± 0.7 DADs/min.; KO iPSC-CM control 5.4 ± 1.2 DADs/min.; KO iPSC-CM + PF-01247324: 4.1 ± 1.2 DADs/min.; Figure 4a,b). The application of PF-01247324 had no significant effect on Na_V_1.8 KO lines, which showed an already reduced incidence of arrhythmic events. Thus, genetic KO or inhibition of Na_V_1.8 markedly prevented cellular arrhythmias in human ventricular CM. We did not observe any early afterdepolarizations in control or Na_V_1.8 KO iPSC-CM.

## 3. Discussion

In this study, we employed CRISPR/Cas9 KO technology in human ventricular CM to demonstrate that the genetic KO of Na_V_1.8 resulted in a substantial reduction in I_NaL_. No significant effects on APD were observed. The heightened frequency of spontaneous arrhythmogenic SR Ca^2+^ sparks, and Ca^2+^ waves elicited by Iso was significantly diminished through pharmacological intervention or genetic KO of Na_V_1.8. Additionally, DADs and spontaneous action potentials, indirectly elicited by an increased SR Ca^2+^ leak, were effectively mitigated through either pharmacological intervention or genetic KO of Na_V_1.8. 

### 3.1. Role of Na_V_1.8 on I_NaL_ Generation

In the current study, we validated the involvement of Na_V_1.8 in Iso-augmented I_NaL_ in human control ventricular iPSC-CM by observing a reduction in I_NaL_ through the specific Na_V_1.8 blocker PF-01247324 or genetic ablation. Our data support the findings of other studies including a report by Yang et al. (2012) [28]. These authors inhibited *SCN10A* channels with a specific Na_V_1.8 blocker in adult *SCN10A-/-* mice and rabbit ventricular CM. Their results showed a reduction in I_NaL_ and anti-arrhythmic effects on ventricular arrhythmias after pharmacologic inhibition of Na_V_1.8 [28]. In our earlier work, Dybkova et al. (2018) identified Na_V_1.8 in human failing ventricular myocardium and demonstrated its significant contribution to I_NaL_ formation [23]. We also exhibited successful modulation of I_NaL_ by Na_V_1.8 inhibitors [23]. Additionally, in another study, Na_V_1.8 expression was significantly upregulated in human left ventricular hypertrophy (LVH), and increased Na_V_1.8 expression was associated with enhanced I_NaL_, potentially contributing to the arrhythmogenic substrate and impaired cardiac function observed in LVH [20]. Of note, in addition to its demonstrated presence in atrial and ventricular human CM (adult and iPSC-CM), Na_V_1.8 associated I_NaL_ has also been characterized in various other species (murine, canine and porcine models); thus, some species- and model-dependent differences should be considered [9,10,26,27,29,30,31,32,33,34,35,36,37,38,39,40,41,42,43,44,45,46,47,48,49,50,51,52,53,54,55,56]

Most of the previous work regarding Na_V_1.8-related characteristics of I_NaL_ was obtained on the basis of pharmacological interventions. Drugs may have additional, unspecific effects, leaving the mechanism by which Na_V_1.8 generates I_NaL_ unclear. The present study addresses this gap in knowledge and confirms our previous results in human *SCN10A*-KO iPSC-CM by demonstrating that Na_V_1.8 plays a significant role in generating I_NaL_ by taking an alternative approach that is not purely pharmacological. Furthermore, our results demonstrate that Na_V_1.8-induced I_NaL_ is not limited to ganglia, as assumed in the past, but is also present in ventricular CM, as shown by the results of our previous project [22].

Since I_NaL_ is typically very small under healthy or basal conditions, we employed isoproterenol to enhance I_NaL_ in our cellular model in order to facilitate a comparison between control and KO iPSC-healthy-CM. It is worth noting that without beta-adrenergic stimulation or structural disease, Casini et al. (2019) did not detect any Na_V_1.8-dependent I_NaL_ in non-diseased rabbit ventricular CM by using specific blockers [57]. This underscores the dependence of Na_V_1.8-associated I_NaL_ enhancement on stimulation by pharmacological (beta-adrenergic activation) or pathological circumstances (e.g., chronic CAMKII_δc_ overexpression [22]), which likely explains the absence of Na_V_1.8 effects in their study. The fact that I_NaL_ is only enhanced under pathological conditions has to be taken into account in the interpretation of experiments and establishes I_NaL_ as a disease-specific target.

### 3.2. Significance of Na_V_1.8 in I_NaL_ and Arrhythmogenesis

Previous studies have established the significant role of I_NaL_ in determining the duration of APs in ventricular CM [27,45,58,59,60,61,62]. Considering that Na_V_1.8 expression is upregulated in human HF, we investigated the impact of Na_V_1.8-induced I_NaL_ on various AP parameters. Consistent with our previous findings, our current study demonstrates that Na_V_1.8 has only minimal effects on ventricular AP parameters in ventricular control CM or in *SCN10A*-KO iPSC-CM. At first glance, the results seem to contradict previous findings regarding ventricular Na_V_1.8 channels, their effect on APD, and their contribution to arrhythmias in the context of HF and hypertrophy. In the failing human heart and in a mouse model with a genetic *SCN10A* KO, Dybkova et al. (2018) demonstrated that Na_V_1.8 expression was higher and that this was associated with a minimally prolonged APD, contributing to I_NaL_-induced arrhythmias [23]. The contribution of Na_V_1.8 to Na^+^- and Ca^2+^-dependent cellular arrhythmias revealed that Na_V_1.8 channels can promote abnormal electrical activity and Ca^2+^ overload in CM. These proarrhythmic effects were mediated by enhanced Na^+^ and Ca^2+^ influx through Na_V_1.8 channels [21]. Moreover, the interaction between CaMKII_δc_ and Na_V_1.8 in HF leads to CaMKII_δc_ phosphorylation of Na_V_1.8 channels, exacerbating the proarrhythmic effects in the failing hearts [22]. In human LVH, Ahmad et al. (2019) demonstrated that enhanced Na_V_1.8 function was associated with AP prolongation [20]. Overall, these studies suggest that the increased expression and activity of Na_V_1.8 channels in failing hearts and hypertrophied ventricles, in contrast to the healthy CM investigated in the present study, may contribute to APD prolongation and proarrhythmic effects. Thus, further investigation of Na_V_1.8 and its inhibition and of the characteristics of I_NaL_ in injured or diseased human iPSC-CM models will be of interest to help clarify mechanisms that play a role under pathological conditions.

We show that the inhibition of Na_V_1.8 by PF-01247324 of I_NaL_ or Vmax was absent in Na_V_1.8 KO ventricular iPSC-CM. Given that Vmax (dv/dt) serves as an indicator of the rapid influx of Na^+^ and the peak Na^+^ current, these findings indicate that Na_V_1.8 does not play a relevant role in the peak Na^+^ current in ventricular iPSC CM.

The association between enhanced I_NaL_ and an increased susceptibility to arrhythmias is intricate [3,9,45]. This complexity stems from the increased leakage of Ca^2+^ from the SR, which can instigate a transient inward current, subsequently giving rise to arrhythmogenic DADs. In the current study, we observed a decrease in the frequency of spontaneous SR Ca^2+^ sparks and spontaneous Ca^2+^ waves in human ventricular iPSC-CM with *SCN10A* KO as well as in control CM after pharmacological inhibition of Na_V_1.8. Since Ca^2+^ waves are known to act as potent proarrhythmic triggers, our findings provide evidence for the involvement of Na_V_1.8-induced I_NaL_ in cellular arrhythmogenesis in isolated human ventricular iPSC-CM. This is in line with our previous investigations, which demonstrated that the I_NaL_, mediated by the influx of Na^+^, can induce the influx of Ca^2+^ via the reverse mode of the sodium-calcium exchanger (NCX) in the human ventricle. This process can lead to an elevation in the cytosolic concentration of Ca^2+^ and an increased occurrence of ryanodine receptor (RyR_2_) Ca^2+^ release events [21]. Furthermore, inhibiting I_NaL_ by using specific Na_V_1.8 channel inhibitors has been shown to reduce the reverse mode of the NCX and, consequently, mitigate diastolic proarrhythmogenic SR Ca^2+^ leakage [20,21,23]. Interestingly, we did not observe any effects on intracellular Ca^2+^ transients in *SCN10A-*KO CM or following Na_V_1.8 inhibition in control CM. This implies there are no negative inotropic effects of Na_V_1.8 inhibition, which is particularly noteworthy considering the situation in HF patients. 

Since I_NaL_-dependent diastolic SR Ca^2+^ release has the potential to induce an NCX-mediated depolarizing current (I_Ti_), resulting in cellular arrhythmias [63,64,65], we examined the impact of Na_V_1.8 on afterdepolarizations and spontaneous action potentials. In our current investigation, a substantial number of DADs was noted in control iPSC-CM following Iso application. This effect was significantly mitigated by either inhibiting Na_V_1.8 with PF-01247324 or through genetic depletion. 

It is widely recognized that an augmented I_NaL_ results in cellular Na^+^ accumulation [66,67], subsequently leading to Ca^2+^ overload through activation of the NCX reverse mode [9,21,26,68,69]. Our earlier investigations have elucidated that this sequence of events may initiate the diastolic release of Ca^2+^ from the SR and the subsequent occurrence of DADs. These effects are attributed to an intensified phosphorylation of RyR_2_, regulated by CaMKII_δc_ [27]; this may explain our current findings and how Na_V_1.8 influences arrhythmogenesis via I_NaL_. 

### 3.3. Clinical Relevance

Ventricular arrhythmias, such as ventricular tachycardia and ventricular fibrillation, are life-threatening conditions associated with a high risk of sudden cardiac death [60,70]. Studies have shown that increased Na_V_1.8-dependent I_NaL_ plays a role in the development of arrhythmia in various pathological conditions, including HF, LVH, and other structural heart diseases in ventricular CM [20,22,23]. Unfortunately, in HF patients, current anti-arrhythmic medications, such as amiodarone, have shown severe adverse effects, potentially including potentially organ toxicity [71]. Therefore, there is a crucial need for safer and more effective compounds to treat arrhythmias, especially in patients with HF. In the context of trials testing oral selective inhibitors in patients, the findings of the aforementioned article suggest a promising and easily implemented strategy for drug repurposing [71]. Targeting Na_V_1.8 and specifically inhibiting I_NaL_ emerges as a potential therapeutic approach to prevent or treat ventricular arrhythmias. The current study shows that either genetic elimination of Na_V_1.8 through *SCN10A* KO in iPSC-CM or pharmacological inhibition of Na_V_1.8 can effectively reverse the proarrhythmic effects at the cellular level in ventricular cells. 

## 4. Materials and Methods

All procedures conducted in this study adhered to the principles outlined in the Declaration of Helsinki and received approval from the local ethics committee of the University Medical Center of Göttingen (Az-10/9/15). Informed consent was signed by all tissue donors.

### 4.1. Generating Homozygous Knockout iPSC

The generation of the homozygous *SCN10A*-KO iPSC line was carried out following the previously described protocols [22,25]. Briefly, we used a previously described wildtype iPSC line [72] as a template and edited it with the CRISPR-Cas9 technique as described in Maurer et al. [25]. The wildtype iPSC were electroporated with a mixture of two guideRNAs, a tracrRNA, and the Cas9 protein using the Human Stem Cell Nucleofector Kit (Amaxa VPH-5022) and the Amaxa Nucleofection II Device (Lonza, program B-016). An electroporation enhancer was added to enhance electroporation efficiency. After electroporation, 72 colonies were expanded, and genomic DNA was analyzed by Sanger sequencing. A promising iPSC mixed clone was chosen for further singularisation. After successful singularisation and additional Sanger sequencing of genomic DNA, two homozygously edited *SCN10A* knockout iPSC clones were obtained. These clones were regularly sequenced to check for maintenance of the knockout gene editing.

### 4.2. IPSC-Cardiomyocyte Differentiation

Two Na_V_1.8 KO iPSC lines (K62.1 and K62.4) and the corresponding isogenic control iPSC line [72] were cultured and differentiated into ventricular iPSC-CM as described previously [22,72]. In brief, iPSCs were cultured in a monolayer on Geltrex^®^-coated dishes in chemically defined E8 medium (Life Technologies) until 80–90% confluency. Then, we initiated cardiac differentiation by manipulating Wnt signaling. Wnt signaling was activated by a medium change to cardio differentiation medium (RPMI 1640 with GlutaMAX medium (Gibco) supplemented with 0.02% L-ascorbic acid 2-phosphate (Sigma-Aldrich) and 0.05% albumin (Sigma-Aldrich)), supplemented with the GSK3 inhibitor CHIR99021 (4 µmol/L, Millipore). Subsequently, Wnt signaling was inhibited by changing the medium 48 h later to cardio differentiation medium supplemented with 5 µmol/L of the inhibitor of Wnt production-2 (IWP2, Millipore) for another 48 h. The medium was changed to cardio differentiation medium without adding small molecules for 48 h. From day 6 onward, the medium was changed to cardio culture medium (RPMI 1640 with GlutaMAX medium supplemented with 2% B27 with insulin (Gibco)), which was changed every 2 to 3 days. From day 14 to 21, the cells began beating. They were then passaged as 600,000 cells/well onto 6-well culture dishes and metabolically selected for CM for 4 to 5 days by depletion of glucose but supplementation of lactate (4 mmol/L) in a specified cardio selection medium (RPMI 1640 w/o glucose supplemented with 0.02% L-ascorbic acid 2-phosphate (Sigma-Aldrich) and 0.05% albumin (Sigma-Aldrich)). IPSC-CM were matured in cardio culture medium, with a medium change twice a week. The purity of the cells was determined by flow cytometry (>90% cTNT+ cells), immunofluorescence staining, and qPCR analysis for cardiac ventricular sub-type markers. Measurements were performed roughly 60 to 90 days after the initiation of the differentiation process. For functional experiments, iPSC-CM were plated on glass dishes with different cell numbers (World Precision Instruments).

### 4.3. Standard Operating Procedures

All electrophysiological measurements were conducted following a standardized protocol. A total of 35,000 ventricular iPSC-CM were plated on glass-bottomed Fluoro Dishes. Spontaneously beating ventricular iPSC-CM were stimulated at 0.5 Hz during experiments to achieve a steady state. Single-cell measurements were performed at room temperature with a stimulation frequency of 0.5 Hz/1 Hz/2 Hz. To selectively inhibit sodium currents induced by Na_V_1.8, a specific Na_V_1.8 blocker, PF-01247324 (1 µmol/L, Sigma-Aldrich, Taufkirchen, Germany), was utilized. Cellular electrophysiological measurements were carried out with mild beta-adrenergic stimulation using isoproterenol (Iso) at a concentration of 50 nmol/L (Sigma-Aldrich, Taufkirchen, Germany). iPSC-CM were incubated with either Iso (50 nmol/L, or Iso + PF-01247324 (1 µmol/L, Sigma-Aldrich, Taufkirchen, Germany) for 15 min before starting measurements. Regarding the specificity of Na_V_1.8 blockers, there are already published data that have demonstrated the specificity of PF-01247324 and showed that it is an efficacious inhibitor of Na_V_1.8 [21,22,73,74].

### 4.4. Patch-Clamp-Experiments

Patch-clamp experiments were performed as previously described [21,22,74,75]. For action potential recordings, we employed the whole-cell patch-clamp technique in the current clamp configuration. The microelectrodes (3–5 MΩ) were filled with the following solution (in mmol/L): 92 K-aspartate, 48 KCl, 1 Mg-ATP, 10 HEPES, 0.02 EGTA, 0.1 GTP-Tris, and 4 Na_2_-ATP (pH 7.2, adjusted with KOH). The bath solution consisted of the following components (in mmol/L): 140 NaCl, 4 KCl, 1 MgCl_2_, 2 CaCl_2_, 10 glucose, and 10 HEPES (pH 7.4, adjusted with NaOH). Action potentials were continuously elicited by square current pulses with an amplitude of 1–2 nA and a duration of 1–5 ms at a frequency ranging from 0.5 to 2 Hz. The access resistance typically ranged from ~5 to 15 MΩ after patch rupture. Fast capacitance was compensated for in a cell-attached configuration, while membrane capacitance and series resistance were compensated after patch rupture. Signals were filtered using 2.9 and 10 kHz Bessel filters and recorded with an EPC10 from HEKA Elektronik Dr. Schulze GmbH, Lambrecht, Germany. Both the measuring electrode and the bath electrode (earth conductor of the bath solution) were made of chlorinated silver wire (HEKA Elektronik Dr. Schulze GmbH, Lambrecht, Germany).

To measure the I_NaL_ ruptured patch, whole-cell patch-clamping was performed at room temperature. The resistance of the pipette was between 2 and 3 mega-Ohm (MΩ) when filled with pipette solution containing (in mmol/L): 95 CsCl, 40 Cs-glutamate, 10 NaCl, 0.92 MgCl_2_, 5 Mg-ATP, 0.3 Li-GTP, 5 HEPES, 0.03 niflumic acid (to block Ca^2+^-activated chloride current), 0.02 nifedipine (to block Ca^2+^ current), 0.004 strophanthidine (to block Na^+^/K^+^ ATPase current) 1 EGTA, and 0.36 CaCl_2_ (free[Ca^2+^]i, 100 nmol/L) (pH 7.2 with CsOH at room temperature). iPSC-CM were maintained in the bath solution containing (in mmol/L): 135 NaCl, 5 tetramethylammonium chloride, 4 CsCl, 2 MgCl_2_, 10 glucose, and 10 HEPES (pH 7.4 with CsOH at room temperature). To minimize contaminating Ca^2+^ currents during I_NaL_ measurements, Ca^2+^ was omitted from the bath solution. I_NaL_ was measured only in those iPSC-CM where a seal of more than 1 giga-Ohm was achieved, and the access resistance remained < 7 MΩ. When the whole-cell patch configuration was achieved, iPSC-CM were given a period of 3 min to be stabilized before conducting measurements. Thereafter, iPSC-CM were held at −120 mV before depolarization to −35 mV for a duration of 1000 ms with 10 pulses and a basic cycle length of 2 s. I_NaL_ was measured as integral current amplitude between 100 and 500 ms and was normalized to the membrane capacitance.

### 4.5. Confocal Ca^2+^ Imaging and Epifluorescence Microscopy

Ca^2+^ imaging and epifluorescence microscopy were performed as previously described [74].

In detail, for confocal Ca^2+^ imaging, ventricular iPSC-CM plated on glass-bottom FluoroDishes were incubated with the Ca^2+^ indicator Fluo 4-AM (10 µmol/L, Invitrogen) for 15 min at room temperature for de-esterification of the dye. The solution was substituted with Tyrode’s solution and the respective pharmacological agents and incubated for 15 min. Confocal line scans were obtained with a laser scanning confocal microscope (LSM 5 Pascal, Zeiss). Scans were conducted after continuous electrical field stimulation at 0.5 Hz during a pause in stimulation. Ca^2+^ release events were analyzed using the SparkMaster plugin for ImageJ. The mean Ca^2+^ spark frequency was calculated from the number of sparks normalized to scan width, duration, and scan rate (100 µm/s). Cells exhibiting major Ca^2+^ release events (Ca^2+^ wavelets or waves) were excluded from the calculation of Ca^2+^ spark frequency and separately classified as proarrhythmic cells as a proportion of all cells.

For epifluorescence microscopy, ventricular iPSC-CM were dissociated and plated as described above and loaded with the ratiometric Ca^2+^ indicator Fura 2-AM (5 µmol/L, Invitrogen) for 15 min at RT. Subsequently, cells were washed with Tyrode’s solution for de-esterification and incubated with pharmacological agents as described above. Measurements were performed using a fluorescence detection system (IonOptix) connected to an inverted microscope with oil immersion lens (40×). CM were subjected to electrical field stimulation at 0.5 Hz for the duration of the experiment to ensure steady intracellular Ca^2+^ concentration. Recording of Ca^2+^ transients for analysis was performed at 0.5 Hz at steady state. The stimulation was paused for 30 s to evaluate the spontaneous beating frequency of iPSC-CM (Supplemental Figure S3). Ca^2+^ transients were analyzed using the software IonWizard (IonOptix).

### 4.6. Statistical Analysis

The data are reported as mean ± SEM unless otherwise stated. Analysis was carried out with Prism 9 software (Graphpad, San Diego, CA, USA). Three or more groups, including more than one differentiation experiment, were compared using nested one-way ANOVA to account for potential variance between iPSC-CM differentiations. The results were corrected for multiple comparisons by Sidak’s correction. Fisher’s exact test was used to statistically compare proportions. P values are two-sided and considered statistically significant if *p* < 0.05. 

## 5. Limitations

Using an iPSC-CM model, it is not possible to discriminate between specific cell types located at different parts of the ventricle (epi-, mid-, endocardial). All experiments were performed on a single-cell level, limiting a direct translation of our results and conclusions regarding arrhythmias into an in-vivo situation (e.g., no electrical cell-to-cell conduction).

## 6. Conclusions

In conclusion, our study demonstrated that Na_V_1.8 is involved in the formation of I_NaL_ in human ventricular iPSC-CM under pathological conditions. Genetic knockout or pharmacological inhibition of Na_V_1.8 effectively modulated proarrhythmogenic triggers, including I_NaL_, diastolic SR Ca^2+^ leak, Ca^2+^ waves, and DADs in human ventricular CM. 

## Data Availability

The original contributions presented in the study are included in the article/Supplementary Materials, further inquiries can be directed to the corresponding authors.

## Supplementary Materials

The following supporting information can be downloaded at https://www.mdpi.com/article/10.3390/ijms25116144/s1.
