# Supplementary material for: NaV1.8 as Proarrhythmic Target in a Ventricular Cardiac Stem Cell Model
Source: Int J Mol Sci. 2024 Jun 2;25(11):6144. doi: 10.3390/ijms25116144 (PMC11172914; doi:10.3390/ijms25116144)
Supplement: Supplementary file 1 [file ijms-25-06144-s001.zip › ijms-2923048-supplementary.pdf]

International Journal of
Molecular Sciences

MDPI

Supplementary Materials

Nav1.8 as Proarrhythmic Target in a Ventricular Cardiac Stem Cell Model

Nico Hartmann [1,2,*,†], Maria Knierim [2,3,†], Wiebke Maurer [1,2], Nataliya Dybkova [1,2], Florian Zeman [4], Gerd Hasenfuß [1,2], Samuel Sossalla [1,2,5,‡] and Katrin Streckfuss-Bömeke [1,2,6,*,‡]

Supplementary Materials

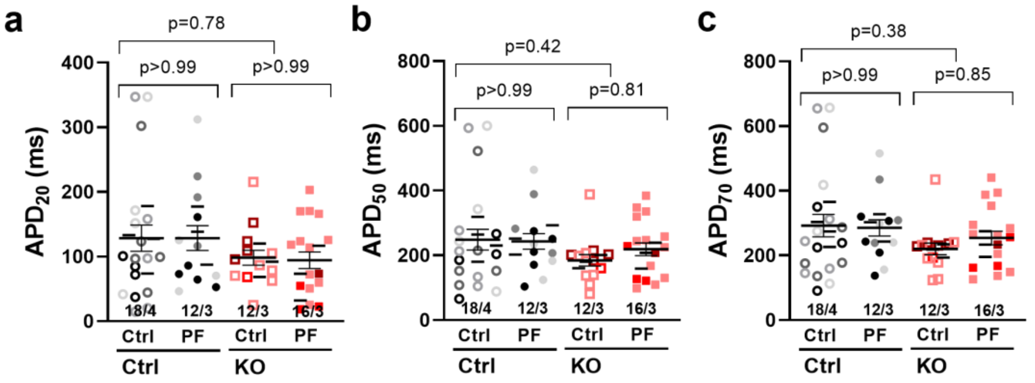

Figure S1: Action potential duration at different stages of repolarization. *The following figures are all shown at* ***0.5 Hz****. Mean values ± SEM of (****a****) APD_{20}, (****b****) APD_{50} and (****c****) APD_{70} of control and SCN10A-KO hiPSCM (control n=18 cells/4 differentiations; control + PF-01247324 [PF] n=12 cells/3 differentiations; SCN10A-KO control n=12 cells/3 differentiations, SCN10A-KO + PF n=16 cells/3 differentiations). Single cells are presented as individual symbols, different iPSC-CM differentiations are colour-coded. The small horizontal lines indicate the mean values per differentiation. Data were compared using nested one-way ANOVA with Sidak's test for multiple comparisons to calculate P values.*

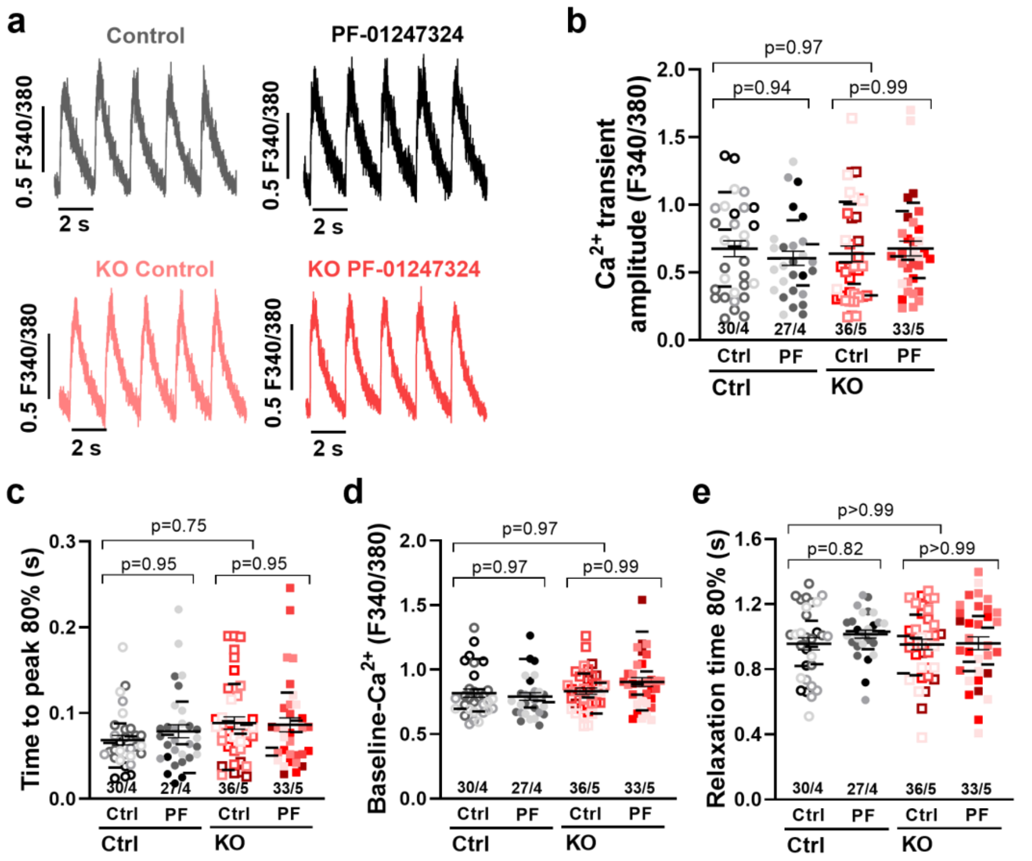

Figure S2: Ca²⁺ transient parameters in ventricular control and SCN10A KO iPSC-CM. (a) *Representative original recordings of stimulated systolic Ca²⁺ transients (epifluorescence microscopy, Fura 2-AM, 0.5 Hz) of human ventricular SCN10A or control iPSC-CM and after additional Nav1.8 inhibition by PF-01247324. Mean values ± SEM of* ***(b)*** *systolic Ca²⁺ transient amplitude,* ***c*** *time to peak 80%, (****d****) diastolic Ca²⁺ level and (****e****) relaxation time 80% in control CM (n=30 cells/4 differentiations), SCN10A-KO CM (n=36/4), and each after treatment with PF-01247324 (control+PF-01247324 n=27/4, KO+PF-01247324 n=33/5). Values are presented as mean ± SEM. Single cells are presented as individual symbols, different iPSC-CM differentiations are colour-coded. The small horizontal lines indicate the mean values per differentiation. Data were compared using nested one-way ANOVA with Sidak's test for multiple comparisons to calculate P values.*

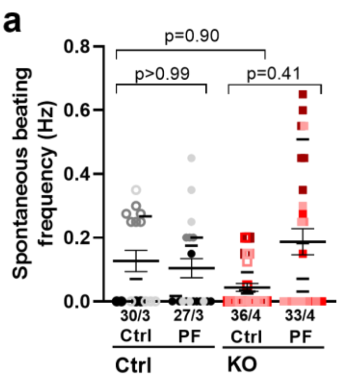

Figure S3: Spontaneous beating frequency of ventricular iPSC-CM. (a) *Mean values of spontaneous beating frequency (bpm) of ventricular iPSC-CM (control or Nav1.8 KO, each with or without Nav1.8 inhibition by PF-01247324). The beating frequency was assessed by measuring spontaneous Ca²⁺ transients with paused stimulation. Single cells are presented as individual symbols, different iPSC-CM differentiations are colour-coded. The small horizontal lines indicate the mean values per differentiation. Data were compared using nested one-way ANOVA with Sidak's test for multiple comparisons to calculate P values.*

Table S1: Action potential duration of ventricular iPSC-CM at 0.5 Hz.

	**APD_{20}**	**APD_{50}**	**APD_{70}**
control	128.4±24.6	248.2±39.1	291.1±40.3
control + PF	127.1±23.5	241.4±29.9	285.5±17.6
SCN10A KO control	99.5±14.1	185.5±22.2	236.9±29.7
SCN10A KO + PF	98.2±14.6	222.0±22.9	270.9±28.7

Table S2: Action Potential Parameters of ventricular iPSC-CM at 1 and 2 Hz.

**Action Potential parameters**	**frequency**	**1 Hz**				**2 Hz**			
		Ctrl./Ctrl.	Ctrl/PF	KO/Ctrl.	KO/PF	Ctrl./Ctrl.	Ctrl/PF	KO/Ctrl.	KO/PF
	RMP (mV)	-76.0	-79.1	-72.0	-81.1	-71.1	-71.2	-70.0	-80.2
	APA (mV)	122.2	127.2	119.3	130.3	118.0	125.4	104.1	129.0
	dV/dt_{max} (mV/ms)	98.2	103.2	114.5	137.9	110.9	118.8	76.3	140.6
	APD_{10} (ms)	80.6	79.8	40.6	43.8	38.8	58.9	18.9	42.9
	APD_{20} (ms)	154.7	152.9	103.3	108.0	91.1	107.6	39.7	107.6
	APD_{30} (ms)	204.2	211.6	142.2	157.2	131.3	156.2	60.1	155.1
	APD_{40} (ms)	236.7	248.4	166.9	190.6	158.4	188.4	74.8	185.2
	APD_{50} (ms)	253.9	279.3	184.8	214.3	180.6	109.7	86.9	207.0
	APD_{60} (ms)	276.0	295.4	200.5	235.5	194.6	227.3	100.3	226.8
	APD_{70} (ms)	300.3	321.6	217.7	252.7	213.3	246.4	116.5	249.6
	APD_{80} (ms)	335.7	370.1	242.6	296.7	234.2	270.2	153.2	260.6
	APD_{90} (ms)	441.9	481.9	324.5	440.2	282.2	327.1	187.5	294.6
	capacitance (pF)	137.4	155.2	126.4	106.0	118.1	151.4	116.1	116.5
